# Using Inverse Probability Bootstrap Sampling to Eliminate Sample Induced Bias in Model Based Analysis of Unequal Probability Samples

**DOI:** 10.1371/journal.pone.0131765

**Published:** 2015-06-30

**Authors:** Matthew Nahorniak, David P. Larsen, Carol Volk, Chris E. Jordan

**Affiliations:** 1 South Fork Research, Inc., North Bend, Washington, United States of America; 2 Pacific States Marine Fisheries Commission, Corvallis, Oregon, United States of America; 3 Northwest Fisheries Science Center, NOAA-Fisheries, Seattle, Washington, United States of America; University of California, Riverside, UNITED STATES

## Abstract

In ecology, as in other research fields, efficient sampling for population estimation often drives sample designs toward unequal probability sampling, such as in stratified sampling. Design based statistical analysis tools are appropriate for seamless integration of sample design into the statistical analysis. However, it is also common and necessary, after a sampling design has been implemented, to use datasets to address questions that, in many cases, were not considered during the sampling design phase. Questions may arise requiring the use of model based statistical tools such as multiple regression, quantile regression, or regression tree analysis. However, such model based tools may require, for ensuring unbiased estimation, data from simple random samples, which can be problematic when analyzing data from unequal probability designs. Despite numerous method specific tools available to properly account for sampling design, too often in the analysis of ecological data, sample design is ignored and consequences are not properly considered. We demonstrate here that violation of this assumption can lead to biased parameter estimates in ecological research. In addition, to the set of tools available for researchers to properly account for sampling design in model based analysis, we introduce inverse probability bootstrapping (IPB). Inverse probability bootstrapping is an easily implemented method for obtaining equal probability re-samples from a probability sample, from which unbiased model based estimates can be made. We demonstrate the potential for bias in model-based analyses that ignore sample inclusion probabilities, and the effectiveness of IPB sampling in eliminating this bias, using both simulated and actual ecological data. For illustration, we considered three model based analysis tools—linear regression, quantile regression, and boosted regression tree analysis. In all models, using both simulated and actual ecological data, we found inferences to be biased, sometimes severely, when sample inclusion probabilities were ignored, while IPB sampling effectively produced unbiased parameter estimates.

## Introduction

In scientific research, it has been found that a high proportion of reported results are irreproducible, and that many published findings are in reality measures of statistical bias [1]. In ecological research, the potential for selection bias in statistical analysis may arise when sampling design is ignored or improperly accounted for in the analysis of data [2]. Selection bias describes bias resulting from, among other causes, a sample that is not representative of the population of interest [3]. Sample selection bias and flawed statistical analysis of sample data have been identified as sources of irreproducible results [4].

Typically, model based statistical methods are built on the assumption that data are obtained with equal sampling probabilities, though often researchers in ecology, and other fields, necessarily use available data from unequal probability samples. Unfortunately, there are numerous examples where such model based analysis is done without accounting for sampling design [2, 5, 6]. Inference from such analyses is therefore susceptible to bias, unless sample inclusion probability (the probability that a given population element is included in the sample) is independent of the parameter(s) being estimated [7, 8]. If sample inclusion probability is not independent of the parameter(s) being estimated, estimates will be biased toward those sites with lower sample inclusion probability, because these sample units are over-represented in the analysis.

While equal probability samples may be preferred in model based analysis, there are, in many cases, a-priori reasons why unequal probability sampling was the preferred sampling design. In sampling a spatial resource, unequal probability designs are often preferred after considering that some population elements are perceived to be more important than others [3]. Stratified sampling or other forms of non-uniform probabilistic sampling can be used to design samples that efficiently estimate population means and trends [8, 9]. These study designs, when properly applied, can increase precision of population estimates and statistical power to detect population trends, especially when the mean or variance of an attribute varies significantly across a definable attribute [10, 11]. Data from complex, unequal probability sampling plans may be ideal for design based estimation of population means and distributions. Examples of probability sampling includes monitoring by the National Park Service, where design based analyses efficiently track long term status and trend of ecological resources [12]; stratified sampling utilized by the Columbia Habitat Monitoring Program (CHaMP) [13] to monitor status and trends in habitat metrics important for juvenile salmon survival; stratified sampling used by the Environmental Monitoring and Assessment Program (EMAP, http://www.epa.gov/emap/); and stratified sampling conducted for the National Aquatic Resource Survey (NARS, http://www.epa.gov/watertrain/monitoring/nationalsurveys.html).

Design based statistical tools are often used for inference on complex statistical sampling designs [2, 10]. In design based inference, population estimates are made using the probabilities of selection for each sample unit [14]. Design based inference is generally used to estimate population means and trends, rather than model parameters that describe complex relationships between variables. Design based analysis tools, such as available in the R programming language [15] using the spsurvey package [16], account for sample inclusion probabilities, and thus provide status and trend estimation unbiased by unequal inclusion probabilities.

However, in ecology, as in other fields, use of such data beyond the original intent for which the sampling process was defined, may be necessary. Researchers wanting to fit data to complex models understandably want to use available data from unequal probability designs or observational data whose inclusion probabilities are not known, rather than re-do expensive sampling with a sample plan specified to meet assumptions of model based statistical analysis, where a user defined model serves as the basis for inference about population parameters [17]. A wide variety of model based analysis tools are available, such as linear regression, quantile regression, and boosted regression trees. In model based inference, the structure of a model is assumed, and model based statistical tools are used to estimate the unknown parameters of the assumed model using an observed sample [13]. The assumed model may describe complex relationships between variables, if the model adequately describes the population [18]. For example, linear regression has been used to examine temporal changes, across a range of covariates, in spatial density of aspen and conifer stands in City of Rocks Natural Reserve [19].

For some model based statistical tools, there are tools available to account for sampling design in the analysis [20, 21]. To this set of tools and methods, we introduce inverse probability bootstrapping (IPB), which has advantages especially appealing to researchers in ecology, in that it is relatively easy to understand and apply, and that it easily applies to any model based analysis because it transforms a sample, rather than modifying the analysis method, prior to analysis of the data, enabling use of commonly used model based tools without advanced knowledge, or development of, specific methods to account for sampling design within the analysis itself.

Inverse probability bootstrapping (IPB) is a method by which data from an unequal probability sample are transformed into equal inclusion probability data via resampling using the inverse of the original sample inclusion probabilities. A single iteration of the resampling process results in an equal probability sample that may be analyzed using model based tools without violation of the assumption of equal sample inclusion probability, though at a loss of information content. Repeated sampling and analysis of IPB samples ensures negligible loss of information content, while eliminating of the potential for sample selection bias.

Inverse probability bootstrapping is not intended as a replacement to simple random sampling. Indeed, simple random sampling is generally the most powerful tool against biased inference; but in cases where unbiased model based inference requires equal probability sampling, it provides a method to use data from existing unequal probability samples.

## Methods

When unequal probability sampling is used and the researcher cannot assume sample inclusion probabilities are uncorrelated to parameters of interest, it is necessary to give sample units corresponding unequal weights in the analysis [22]. Inverse probability bootstrap (IPB) is an application of sample importance re-sampling [23] applied to probability samples in order to enable unbiased model based parameter estimates from un-equal probability samples.

Bootstrapping is a statistical technique primarily utilized to provide a means of estimating standard errors of statistical estimates [24]. In bootstrapping, re-sampling, with replacement, is done on a sample, and parameter estimates are made from each bootstrap re-sample. The process is repeated thousands of times, and standard errors of parameter estimate(s) are estimated as the standard deviation(s) of the bootstrapped estimates. Additionally, bootstrapping has been applied to analyses of complex probability surveys [25, 26], including techniques where the inverse sample inclusion probabilities are used to generate weighted bootstrap samples.

Typically it is the variation of bootstrapped estimates, rather than mean of the bootstrapped estimates, that is of interest. However, we can also utilize the mean of the bootstrapped estimates as a parameter estimate. We exploit this property of the bootstrap in the analysis of unequal probability samples, where model based tools are not appropriate for the initial probability sample, but are appropriate for a carefully constructed bootstrap sample where the elements of the bootstrap samples have equal sample inclusion probabilities. Thus the basic approach of inverse probability bootstrapping (IPB) is to generate equal probability bootstrap samples by application of unequal weights in the re-sampling state, such that each bootstrap sample is transformed into an equal probability sample. Thus sample inclusion probabilities are handled by modifying the sample itself, rather than through modification of the model based analysis tool. This allows valid inference from model based statistical methods that do not explicitly incorporate survey design into the analysis. Because IPB sampling modifies a sample, rather than an analysis tool, it can incorporate a wide range of model based statistical tools for analysis of unequal probability samples.

Inverse probability bootstrapping can be summarized as seven discrete steps:

1. From an existing unequal probability sample, obtain or calculate sample inclusion probabilities as would be done for any design based analysis, as described in numerous statistical sampling texts [10].
2. Calculate inverse sample probabilities. Let P_*i*,_ be the probability that a given element, *i*, of a sample of size *N*, is included in the original, unequal probability sample. The inverse sample probability assigned to element *i*, P_*i*,*ipb*_, is calculated as follows:
  $${\text{P}}_{i,ipb}=\frac{\frac{1}{{\text{P}}_{i}}}{\sum_{1}^{N}\frac{1}{\sum_{1}^{N}P_{i}}}$$ (1)
  The quantity P_*i*,*ipb*_ is simply the inverse of the original sampling probability, scaled such that the sum of all P_*i*,*ipb*_ is equal to one.
3. Generate an IPB sample. From the original unequal probability sample, re-sample, with replacement, using the inverse sample probabilities P_i,ibp_ calculated using Eq (1). The sample size of the re-sample is equal to the original sample size, N. This step is identical to a single iteration of a conventional bootstrap, except for the use of inverse sampling probabilities rather than uniform re-sample probabilities. As in conventional bootstrapping, sampling *with replacement* is critical. Re-sampling without replacement would result in a re-sample that is identical to the original sample, rather than an equal probability sample. Note that elements of the original sample may be missing from the IPB sample, and other elements from the original sample may be present two or more times in the IPB sample.
4. Conduct the desired model based analysis on the inverse probability bootstrap sample obtained in step 3. Estimate parameters of interest.
5. Iterate steps 3 and 4, as in conventional bootstrapping, and record the parameter estimate(s) from each iteration.
6. Estimate model parameters as the average of parameter estimates over each iteration of the IPB sampling process (steps 3 and 4).
7. Estimate standard errors of parameter estimates as the standard deviation of parameter estimates obtained at each iteration of the bootstrap process, as in conventional bootstrapping. Alternatively, use cross validation methods to assess model precision.

For some model based analysis tools, such as boosted regression tree analysis, averaging parameter results over multiple iterations of a bootstrap analysis may not be practical or possible, since model selection is implicitly bound to the analysis method. While predictions, rather than parameter estimates may be the objective of such analyses, the analysis should still seek unbiased parameter estimates to ensure unbiased model predictions. In these cases, a single inverse probability sample still enables model fitting unbiased by sample inclusion probabilities. However, a single inverse probability bootstrap fails to provide adequate precision, as excessive information content from the original sample is lost in a single iteration of the bootstrap process. Greatly inflating the sample size of the inverse probability bootstrap, rather than iterating, will yield an equal probability sample that does not suffer from significant loss of information content. To inflate the sample size, let N_prob_ be the original size of the sample, and set N_ipb_ to be a value many times greater than this value. For inflated sampling, sample, with replacement, to a sample size of N_ipb_ rather than N_prob_. We refer to the quantity N_ipb_/N_prob_ as the IPB inflation factor. This inflation factor is analogous to the number of iterations of an inverse probability bootstrap required to achieve repeatable results when not using inflated sampling with IPB. Model analysis of the inflated sample will yield unbiased estimates, since the uniform sample inclusion property of the IPB sample is maintained. However, because the sample size is inflated relative to the true sample size, the standard errors from the resultant model fit will be vastly under-estimated, because the sample size used in the model fitting process is much greater than the true sample size. Because using an inflation factor with inverse probability bootstrap sampling drastically underestimates standard errors, model precision should be estimated using another method, such as cross validation. Further work is needed to develop theoretical or corrected bootstrap estimates of standard errors for parameters estimated using inflated inverse probability bootstrap samples. When not using an inflation factor, reported standard errors can be used in conjunction with IPB, as described above.

## Results

To demonstrate biases induced from ignoring sample inclusion probabilities, as well as the effectiveness of inverse probability sampling in eliminating this bias, a series of simulated and example analyses using model based techniques on simulated and actual ecological data were performed. Simulated data were utilized to enable comparisons between known parameter values and parameter estimates obtained from model based statistical techniques. Analyses of actual ecological data were performed to demonstrate that, in real applications, the relationship between sample inclusion probabilities and model parameters is such that failing to account for sample inclusion probabilities does, in practice, result in problematic bias that can be corrected by using inverse probability bootstrapping.

### Simulation 1

#### Inverse Probability Sample: Validation of Inclusion Probabilities

We used stratified sampling on a simulated population to demonstrate that application of IPB sampling on an unequal probability sample does result in a uniform probability sample suitable for analysis via model based analysis tools. We first simulated a population containing 100 elements, labeled 1 to 100, where we defined elements 1–25 as stratum A, 26–50 as stratum B, 51–75 as stratum C, and 76–100 as stratum D. We defined a stratified sample design calling for a sample of 20 total units, stratified such that the sample size per stratum, and resulting inclusion probabilities, were as defined in Table 1. The resulting stratified sample is an example of an unequal probability sample. From the stratified sample, we used IPB sampling to obtain equal probability samples. Our IPB sample size was N = 20 (identical to the initial sample size), and our inverse sample inclusion probabilities were calculated according to Eq 1. For our analysis, the entire simulation process (stratified sampling followed by IPB sampling) was repeated 100,000 times, and sample inclusion probabilities by stratum were averaged overall all repetitions.

**Table 1:** Unequal inclusion probabilities for stratified sample.

Stratum	Total Population Size	Sample Size (N)	Inclusion Probability (P_strat)_
**A**	25	2	2/25
**B**	25	3	3/25
**C**	25	6	6/25
**D**	25	9	9/25
**Total**	100	20	

**Fig 1:**
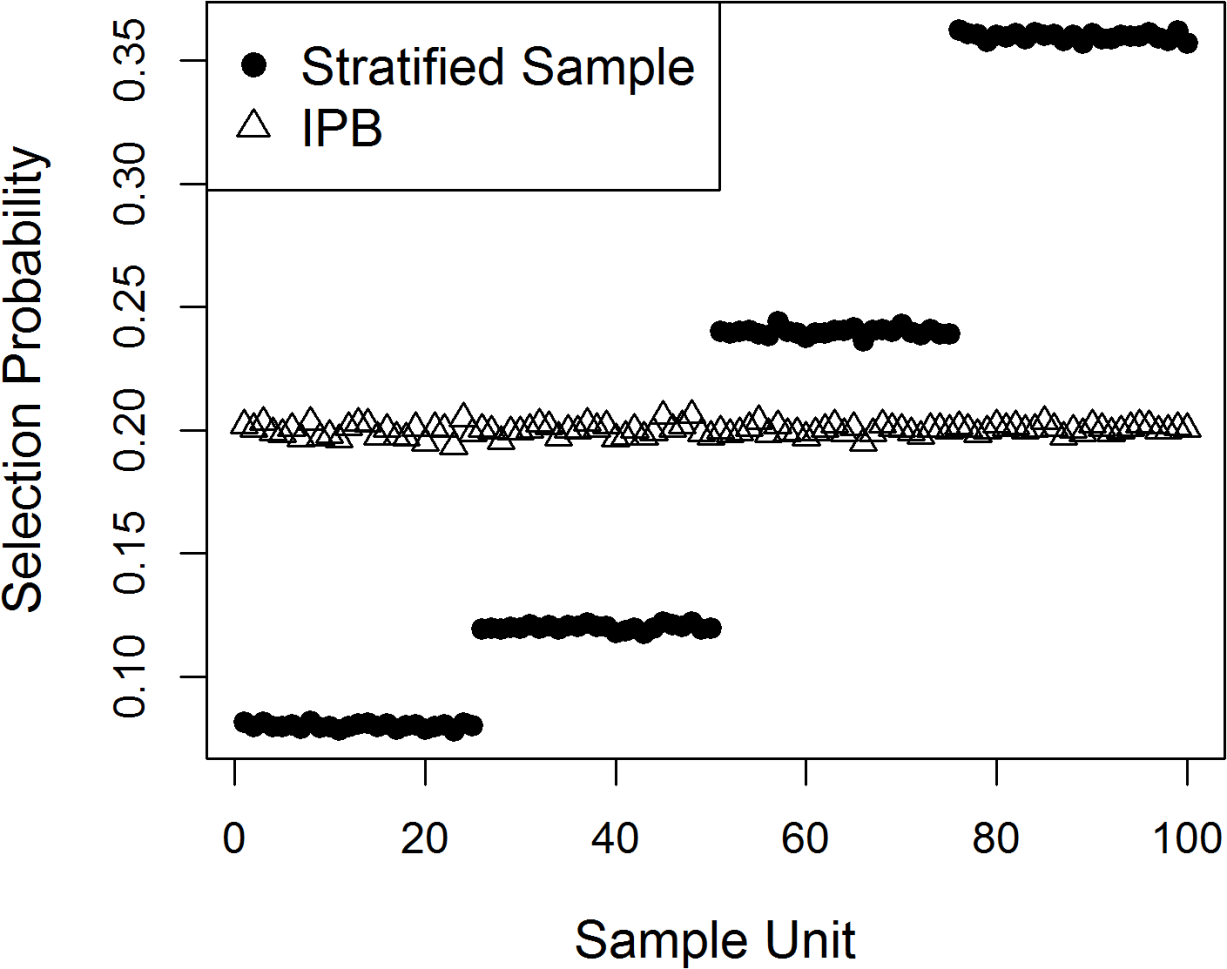
Simulated population sample unit inclusion probabilities for stratified sample and inverse probability bootstrap (IPB) of stratified sample

The number of times each element appeared in the simulated sample, divided by the total number of samples taken over all repetitions of the simulation, estimates the underlying sample inclusion probabilities (Fig 1). As expected, estimated inclusion probabilities for the stratified sample matched the stratified design criteria (Table 1), with approximately 8% for group A (units 1–25), 12% for group B (units 26–50), 24% for group C (units 51–75), and 36% for group D (units 76–100). Inclusion probabilities for the IPB were equal for all sample units, as desired, at 20% for all units. This was as expected given the initial sample size of 20 out of 100 units. This example demonstrates that IPB sampling successfully produces equal probability subsamples from an unequal probability sample, thereby satisfying the equal probability assumption of model based analysis methods.

### Simulation 2

#### Analysis of Simulated Ecological Data

Next, we generated simulated ecological data, then sampled from the simulated dataset using equal probability sampling, stratified sampling, and IPB sampling of the stratified samples. The equal probability sampling (simple random sampling) serves as the ideal, zero bias baseline to which we compare analysis of the stratified samples and the IPB sampling. For each type of sampling, we analyzed the sampled data using three model based statistical tools, the results of which were used to compare bias and standard errors from each of the three sampling techniques.

Data from ecological samples are often comprised of spatially correlated and/or clustered metrics. To generate simulated ecological data, samples for seven simulated variables were generated using the function *cluster*.*Gen* from the R package *clusterSim* [27]. This function allows the user to specify a vector of mean values and a variance-covariance matrix for a series of variables and returns a matrix of partially clustered variables exhibiting the specified mean and variance-covariance structure. For our simulated ecological data, one response variable was arbitrarily considered as the response variable, and six were recorded as explanatory variables. The vector of means and the variance-covariance matrix are shown in Table 2. Note that there are non-zero covariance values, which resulted in simulated populations where the response variable, Y, was positively correlated to the vector of explanatory variables X, and where there was correlation among the explanatory variables.

**Table 2:** Variance-covariance matrix input to R function *cluster*.*Gen* to simulate correlated and clustered explanatory (X1. X6) and response (Y) ecological variables.

		Variance-Covariance Matrix						
Variable	Mean	Y	X1	X2	X3	X4	X5	X6
**Y**	2.0	2.0	0.9	0.5	3.0	0.3	0	0.1
**X1**	4.0	0.9	4.0	0	0.4	0	0	0.5
**X2**	3.0	0.5	0	3.0	0	0	0	0
**X3**	2.0	3.0	0.4	0	7.0	2.0	0	2.0
**X4**	2.0	0.3	0	0	2.0	4.0	0	0.2
**X5**	3.0	0	0	0	0	0	2.0	1.0
**X6**	8.0	0.1	0.5	0	2.0	0.2	1.0	5.0

For each iteration of the simulation, 1400 rows of data were generated. These data represented the total population of interest (the simulated population), where 1400 suggests a large, but finite population from which samples are taken, typical of real world ecological sampling. From the simulated population, 400 rows of data were sampled using three methods. First, a simple random sample (equal probability sample), without replacement, of 400 rows was drawn from the data, using the *sample* function in R. A second sample employed stratified random sampling, where sample inclusion frequencies were defined according to stratum. Strata were defined, arbitrarily, based on the value of the response variable, Y, such that five strata were defined (A–E) along increasing values of Y. Sample inclusion probabilities for each stratum were defined such that the frequency of samples from each stratum in the final sample varied, by up to a factor of three, from the frequency from each stratum in the original population (Table 3). A factor of three is well within the range of sample inclusion probabilities observed in real world ecological sampling (in the real world data example to follow, sample inclusion probability ranges of more than 10x were used). Stratified sampling was performed from the simulated population using the R *sample* function, without replacement. Third, IPB samples were generated from the stratified sample using the IPB process described above. The entire process of sampling, using each of the three combinations of sampling and analysis (simple random sampling, stratified sampling without IPB, and stratified sampling with IPB), was repeated thousands of times, in conjunction with the analysis described below, to generate distributions of parameter estimates and corresponding distributions of errors.

**Table 3:** Simulated population for example 2: distribution by stratum, and target sample size by stratum for stratified sample design.

Stratum	Population Total Count in Stratum	Percent Stratum in Population	Sample Probability Per Unit	Target Sample Size	Percent Stratum in Sample
**A**	435	31%	0.71	311	62%
**B**	183	13%	0.36	65	13%
**C**	182	13%	0.18	33	7%
**D**	143	10%	0.18	26	5%
**E**	457	33%	0.14	65	13%
**Total**	1400	100%		500	100%

For the three sampling and analysis approaches considered in example 2 (simple random sampling, stratified sampling, and IPB analysis of stratified sample data), three sets of model based analyses were conducted: linear regression, quantile regression, and boosted regression tree analysis.

Linear regression models the relationship between a single response variable and one or more explanatory variables. Quantile regression is a model based statistical tool used to estimate quantiles, rather than the mean, of a population response variable as a function of explanatory variables. In ecology, upper quantile estimates of population density are sometimes used as an estimate for species carrying capacity [28]. In other ecological research, quantile regression has been used to draw insight into limiting factors [29, 30]. For all quantile regression analyses in this paper, we modeled the upper 95^th^ percentage quantile. Linear regression and quantile regression both provide direct estimates of model coefficients for each explanatory variable. The presumed true values for each coefficient, to which modeled estimates were compared, were those obtained from linear and quantile regression on all 1400 data points of the simulated population. Boosted regression tree analysis is a technique of predictive data mining that has become increasingly popular in the analysis of ecological data [31]. Potential advantages of boosted regression trees over more traditional regression approaches include the implicit ability to account for interactions and non-linear relationships between variables [32].

To assess bias and the distribution of prediction error, cross validation was incorporated into each analysis. For each of the 1400 initial rows of data in the population, one row at a time was removed from the data set, and 400 data points were sampled from the remaining 1399 data points. From the sample, each model was fit, and the fitted models were used to predict the value of the excluded data point. Prediction error was defined as the predicted value minus the actual value of the response, for each data point in the population. This was repeated for each data point in the population, yielding a distribution of prediction errors for each model.

We calculated bias as the average prediction error for each model and compared it across all three simulated datasets for each model. Standard deviations of the predicted validation points were calculated as an estimate of the model precision. Standard error estimates for each model parameter estimated were calculated, as in conventional bootstrapping, as the standard deviation of the parameter estimates over each iteration of the bootstrap process. Note that standard errors of parameter estimates are a property of the estimator, and are a function of the sample size rather than the number of IPB iterations, thus do not tend toward zero with an increasing number of IPB iterations. The precision of our estimates of bias, however, does increase with greater numbers of bootstrap iterations. Sufficient IPB iterations were used to ensure uncertainties in our estimates of bias are negligible.

Both simple random sampling and IPB sampling achieved unbiased predictions, while stratified samples analyzed without consideration of sample inclusion probabilities resulted in biased predictions (Table 4). Estimated biases were near zero for all modeling methods (linear regression, quantile regression, and cluster analysis) for models built on data from simple random sampling and IPB sampling, with mean errors ranging from -0.02 to 0.00 (Table 4. Note that, because we’re using simulated data, we have not included units). Biases were non-zero for analyses of the stratified sample that ignored sample inclusion probabilities, with estimated biases of 0.30, 0.40, and 0.70 for linear regression, quantile regression, and boosted regression tree analysis, respectively. The precision of predictions, estimated by the standard deviation of prediction errors, was comparable to or only slightly higher for IPB sampling than for simple random sampling, and roughly equal between IPB sampling and analysis of the stratified sample that ignored sample inclusion probabilities (Table 4). Note, however, that judging a model using only standard deviations of predictions from biased estimates may be misleading in that it suggests a false level of confidence. Bias induced by neglecting to include sample inclusion probabilities is not reflected in the standard deviation of predicted values, thus comparable standard deviations cannot, in the presence of bias, be used to suggest two methods are equally valid.

**Table 4:** Bias and standard deviation of predicted values, by sampling design and modeling method, for simulated ecological data in example 2.

	Linear Regression		Quantile Regression		Boosted Regresstion Tree	
	Bias	Std. Dev.	Bias	Std. Dev.	Bias	Std. Dev.
**Simple Random Sampling**	0.00	1.07	0.02	0.23	0.00	1.53
**Stratified Sample, Ignoring Inclusion Probability**	0.30	1.07	0.40	0.39	0.70	1.54
**Inverse Probability Sampling**	-0.02	1.08	-0.02	0.40	0.00	1.53

Bias is calculated as mean prediction error in leave one out cross validation. For quantile regression, point-wise comparisons for cross-validation are approximated using a linear regression model built from all simulated data.

**Fig 2:**
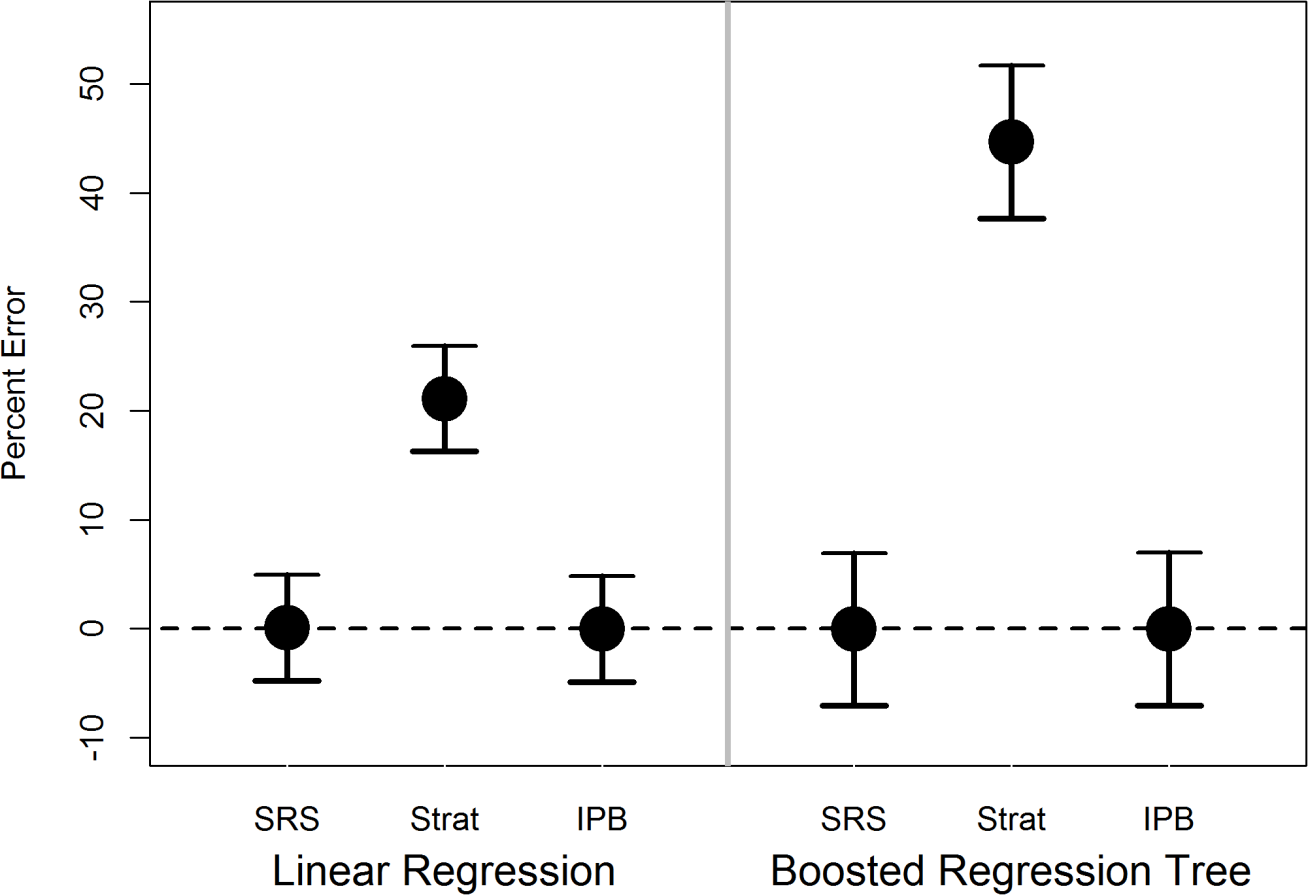
Estimated mean prediction errors, as a percent of the standard deviation of the response variable, with 95% confidence intervals, for linear regression of response variable on independent variables, and boosted regression tree anlaysis, using: simple random samples (SRS), stratified samples fit without accounting for design weights (Strat), and inverse probability bootstrap sampling (IPB)

For linear regression and boosted regression tree analysis, bias was not observed in the results for simple random sampling and IPB sampling, while mean errors were biased (not centered about zero) when sample inclusion probabilities were ignored (Fig 2).

**Fig 3:**
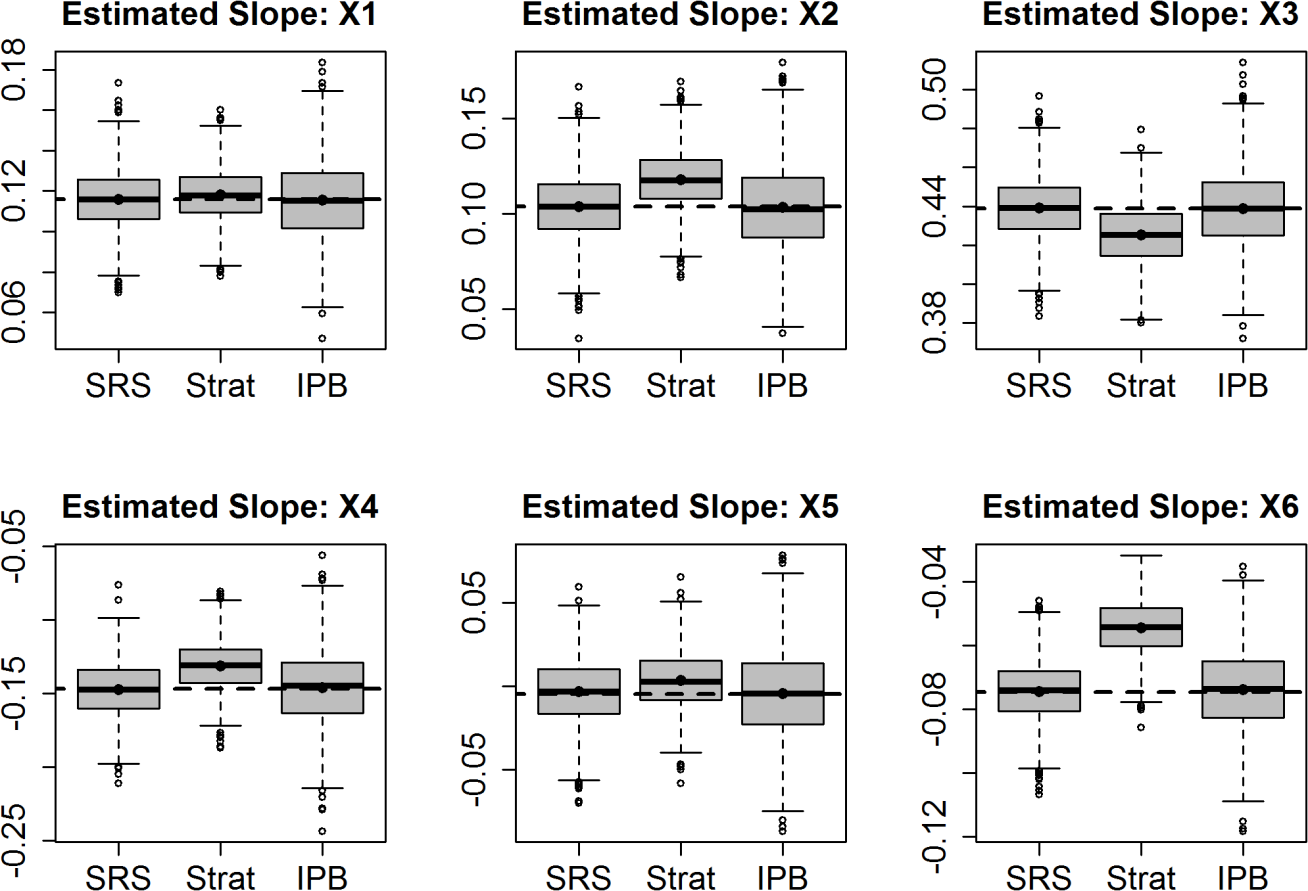
Distribution of estimated slopes for linear regression, using simple random samples (SRS), stratified samples fit ignoring sample inclusion probabilities (Strat), and regression using Inverse Probability Bootstrap samples (IPB)

Bias in estimated coefficients was near zero for both simple random sampling and IPB sampling, with absolute errors, over both models, ranging from zero to 13%, across the estimates for the size model coefficients and intercept (Table 5). Bias is expressed as a percent error between the known true slope and the modeled slope from the sampled data. For analysis of the stratified sample that ignored sample inclusion probabilities, the bias values for the various modeled coefficients ranged from 2% to more than 200% and included biases such as 15%, 24%, and 70% in absolute value. Additionally, there was consistency in the relative slopes between analysis based on simple random sampling and IPB sampling, and the inherent bias present in the analysis of the stratified sample that ignored sample inclusion probabilities (Fig 3).

**Table 5:** Linear model regression results for simulated data. Standard errors refer to the precision of the parameter estimates.

		Simple Random Sample			Stratified Sample: Inclusion Probabilities Ignored in Model Fitting Process			Inverse Probability Bootstrap		
Parameter	True Slope	Estimate Slope	% Error	Std Error	Estimate Slope	% Error	Std Error	Estimate Slope	% Error	Std Error
Intercept	0.153	0.152	0%	0.048	-0.189	-224%	0.045	0.148	-3%	0.056
X1	0.098	0.098	0%	0.015	0.113	15%	0.013	0.098	0%	0.019
X2	0.111	0.110	-1%	0.020	0.124	12%	0.017	0.112	1%	0.025
X3	0.436	0.437	0%	0.015	0.429	-2%	0.014	0.437	0%	0.019
X4	-0.120	-0.120	0%	0.018	-0.110	-9%	0.016	-0.120	-1%	0.024
X5	0.008	0.009	13%	0.019	0.013	70%	0.017	0.007	-10%	0.025
X6	-0.080	-0.080	0%	0.009	-0.057	-24%	0.008	-0.080	0%	0.013

**Fig 4:**
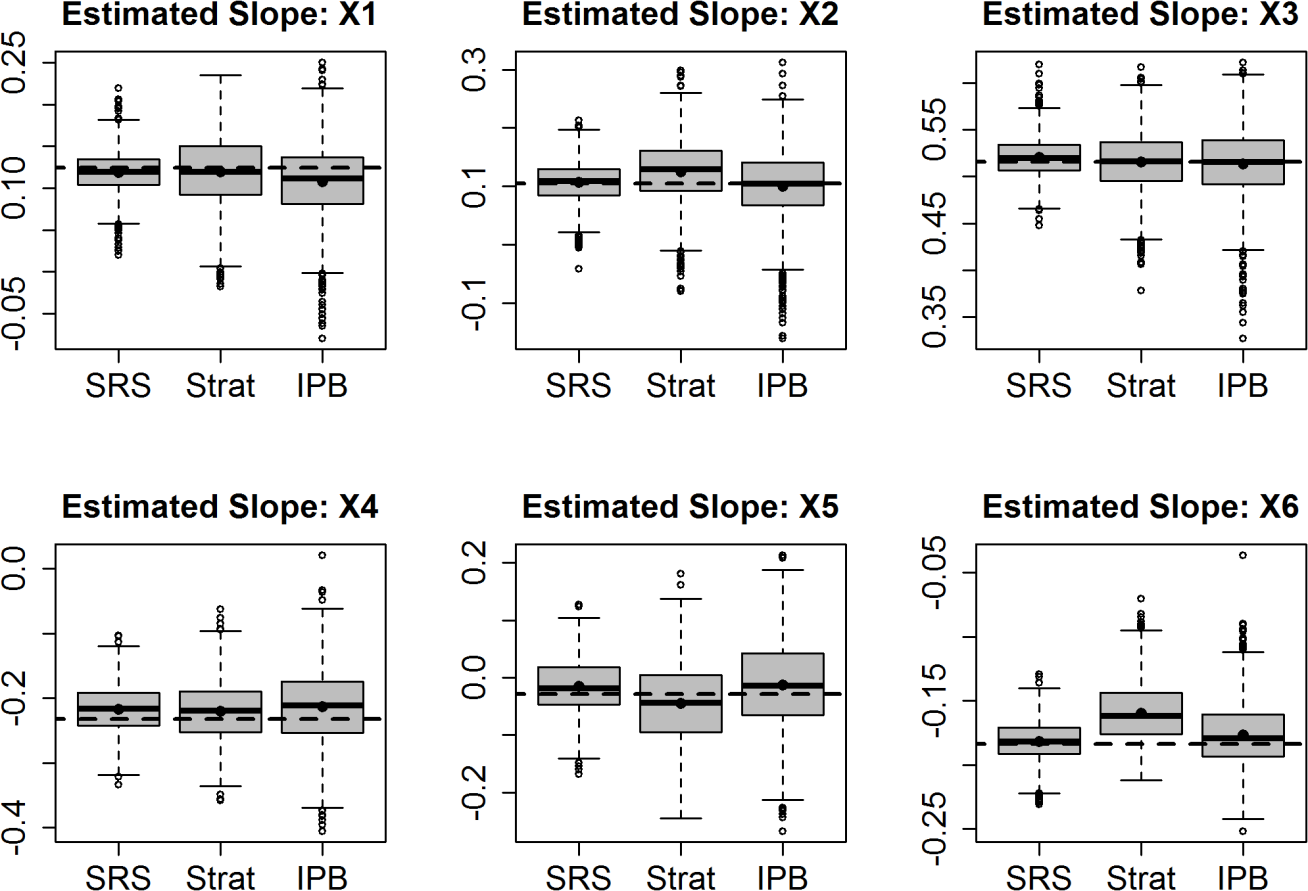
Distribution of estimated slopes for quantile regression, using simple random samples (SRS), stratified samples fit ignoring sample inclusion probabilities (Strat), and regression using Inverse Probability Bootstrap samples (IPB)

Using quantile regression, we again found bias to be present when sample inclusion probabilities were ignored. Errors in estimated coefficients are higher for 5 of 6 coefficients and for the intercept when sample inclusion probabilities were ignored than for estimates made using simple random sampling or IPB sampling (Table 6 and Fig 4).

**Table 6:** Quantile regression results for simulated data. Standard errors refer to the precision of the parameter estimates.

		Simple Random Sample			Stratified Sample: Inclusion Probabilities Ignored in Model Fitting Process			Inverse Probability Bootstrap		
Parameter	True Slope	Estimated Slope	% Error	Std Error	EstimatedSlope	% Error	Std Error	Estimated Slope	% Error	Std Error
Intercept	2.333	2.293	-2%	0.089	1.946	-17%	0.125	2.276	-2%	0.136
X1	0.148	0.133	-10%	0.045	0.129	-13%	0.052	0.124	-16%	0.070
X2	0.123	0.126	2%	0.048	0.140	14%	0.056	0.125	2%	0.073
X3	0.513	0.514	0%	0.039	0.551	7%	0.035	0.504	-2%	0.055
X4	-0.200	-0.205	3%	0.051	-0.234	18%	0.049	-0.202	1%	0.074
X5	0.067	0.050	-24%	0.052	0.004	-94%	0.071	0.030	-54%	0.089
X6	-0.190	-0.190	-1%	0.021	-0.163	-15%	0.023	-0.183	-4%	0.032

**Fig 5:**
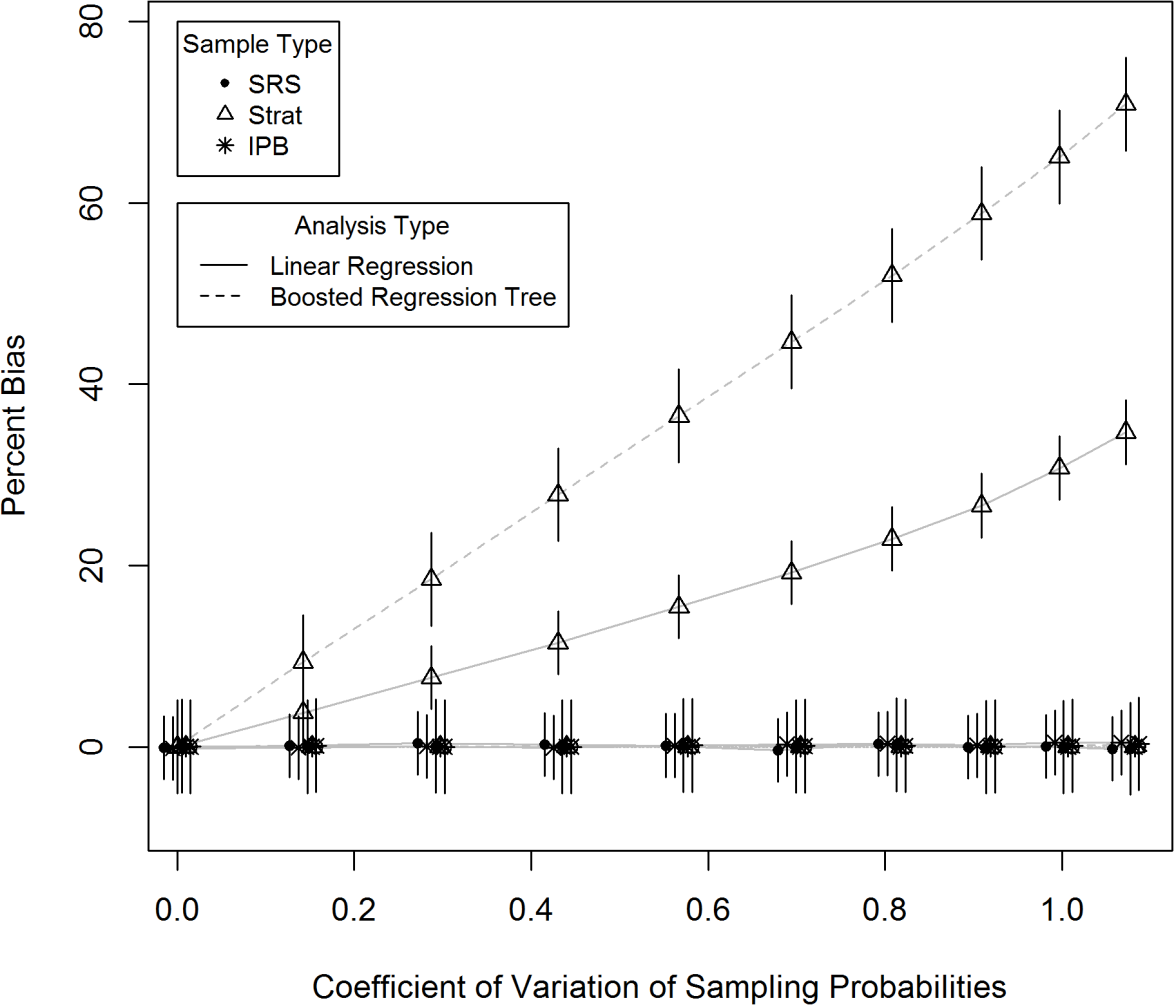
Estimated mean prediction error, as a percentage of the standard deviation of the response variable, for linear regression and boosted regression tree analysis, across a range of variability levels in sample inclusion probability, using simple random samples (SRS), stratified samples fit without accounting for design weights (Strat), and Inverse Probability Bootstrap sampling (IPB).

To further illustrate potential for bias, we repeated the simulation described above at multiple sets of inclusion probabilities. We used the coefficient of variation (COV) as a measure describing variability within the sample inclusion probabilities, where a COV of zero indicates uniform sampling probabilities, and higher COV values describe greater ranges of sampling inclusion probabilities. The same stratification structure was used as in the previous analysis, except that the relative differences in stratum specific sampling probabilities were applied over a wider range. As expected we found that, as the variability in sampling probabilities increased, the amount of bias observed increased (Fig 5). Expressed as a percentage of the standard deviation of the response variable, bias ranged from zero for uniform probability sampling, to more than 60 percent for boosted regression tree analysis, and 40 present for linear regression, at sample probability COV values near 1.0. The sample inclusion probabilities used for the full analysis, described above, had a sample inclusion probability COV of 0.7.

### Example 3. Model Fitting on Real World Ecological Data using Inverse Probability Bootstrapping

The Columbia Habitat Monitoring Program (CHaMP) collects habitat data in watersheds across the interior Columbia River basin (details available on www.champmonitoring.org). The sampling designs for CHaMP are constructed by dividing qualifying networks of rivers and streams into approximately 1 km sites from which a total population frame is defined. From the population frame, a stratified sampling design is used to determine the sampled sites. Within each sampled site, habitat attributes are measured and more than 100 CHaMP metrics are measured for each site.

An Integrated Status Effectiveness Monitoring Program (ISEMP, www.isemp.org) samples juvenile salmonid abundance at a subset of co-located CHaMP sites to estimate the relationship between juvenile abundance and stream habitat attributes. Unique strata and sampling probabilities for sites within each stratum are defined for each sampled site having both fish and habitat data. All strata were defined during the sampling design process, prior to data collection; thus the sample inclusion probabilities for all sampled points can be calculated directly.

In addition to estimating status and trend for habitat and salmonid abundance, an objective of CHaMP / ISEMP research is improved understanding of the complex relationships between stream habitat and salmonid abundance. Model based analysis tools are appropriate and necessary for this objective. While inference within each stratum of each watershed is often useful, there is also interest among researchers and funding agencies to expand understanding of fish habitat relationships across strata within watersheds, as well as across all watersheds over the entire interior Columbia basin. Datasets at spatial scales greater than individual strata contain a range of non-uniform sample inclusion probabilities.

Inverse probability bootstrap sampling was developed in order to support modeling efforts underway in the CHaMP/ISEMP programs. Thus, in order to demonstrate IPB with actual ecological data, we used data from the CHaMP/ISEMP programs, and conducted the same series of model based analyses with this data as were performed with the simulated data.

CHaMP/ISEMP data for 350 sites that contained both fish abundance and habitat data were included from the Entiat, Wenatchee (WA), John Day, Upper Grande Ronde (OR), Lemhi, and South Fork Salmon (ID). Data were collected over three years (2011–2013) as part of a sampling design comprising both annual and rotating panel temporal sampling patterns. Annual sites were sampled each year, and rotating panel sites sampled once in the three years of sampling completed to date. For sites with fish or habitat data sampled at more than one year, site level metrics were averaged across all years. Site level inclusion probabilities were calculated based on the number of sampled sites within each stratum, divided by the total number of sites in the population belonging to that stratum (CHaMP sample design details available on www.monitoringresources.org).

For all analyses, the response parameter is juvenile steelhead density, in fish/m^2^, as measured at each sampled stream location. A subset of CHaMP metrics, believed important to fish abundance, were used as explanatory variables: *conductivity*, *bankfull area*, *wetted large wood volume*, *fast non-turbulent area*, *mean bankfull width*, *percent boulders*, *LWD fish cover*, *discharge*, and *fines >2mm* (see Table 7 for units and definitions). We found that transforming all variables, including steelhead density, with the natural log function, generally resulted in approximately normally distributed model residuals. Note that the analyses presented here are not intended to demonstrate a complete or adequate description of the relationships between habitat and abundance, but merely to demonstrate potential bias when failing to account for sampling design, and the utility of IPB sampling to eliminate such bias.

**Table 7:** Definition and measurement units for CHaMP habitat metrics used in example 3 (www.champmonitoring.org).

Variable	Definition	Units
Conductivity	Measure of the concentration of ionized materials in water, or the ability of water to conduct electrical current	μmhos/cm
Site Bankfull Area	The total bankfull area of a site	m^2^
Wetted Large Wood Volume By Site	Total volume of qualifying large wood pieces (> 0.1 m diameter, 1.0 m length) that touch the wetted perimeter	m^3^
Fast Non-Turbulent Area	Total wetted surface area identified as fast non-turbulent channel units	m^2^
Mean Bankfull Width	Mean bankfull width derived from cross-sections	m
Percent Boulders	Percent of boulders and cobbles within the wetted site area	%
Fish Cover Composition LWD	Percent of wetted area that has large woody debris as fish cover	%
Site Discharge	Volumetric flow rate at site	m^3^/s
Fines <2mm	Average percentage of pool tail substrates comprised of fine sediment <2 mm	%

Just as in the simulation exercise, models from linear regression, quantile regression, and boosted regression tree analysis, were fit two ways, 1) ignoring sample inclusion probabilities; and 2) with IPB sampling to account for sample inclusion probabilities. Unlike the analysis of simulated data, there are not known true parameter values with which to compare parameter estimates. We instead estimate the level of bias by comparing the difference in estimates obtained with and without utilization of inverse probability bootstrapping. Based on the prior discussion and the results of the prior simulations, we assume that the estimates obtained from IPB sampling are indeed unbiased. Estimates of bias are again made from leave-one-out cross validation, where each point is weighted by the inverse sample probability. Note that weighting, during cross validation, is necessary in estimation of bias, since the cross validation points, obtained from the original unequal probability sample, require proper weighting for an unbiased estimate of bias in errors. Failing to account for sample inclusion probabilities during cross validation would otherwise lend itself to bias just as failing to account for sample inclusion probabilities would in the original analysis. In this case, a proper estimate requires only a weighted average of cross validation errors, where the weight for each validation point is equal to the inverse of the initial sample inclusion probability. For quantile regression, there is no corresponding tool for cross validation as there is no point by point estimate of quantile with which to compare predicted quantiles. In this case, analyses of the IPB samples are assumed unbiased, and the quantile regression bias defined for the model that ignores sample inclusion probabilities is taken as the difference in predictions between the estimates from the two sampling methods.

As in the simulated dataset of example 2, parameter estimates from models fit to CHaMP/ISEMP data differed significantly between analyses that ignored sample inclusion probability and models built from IPB samples.

**Fig 6:**
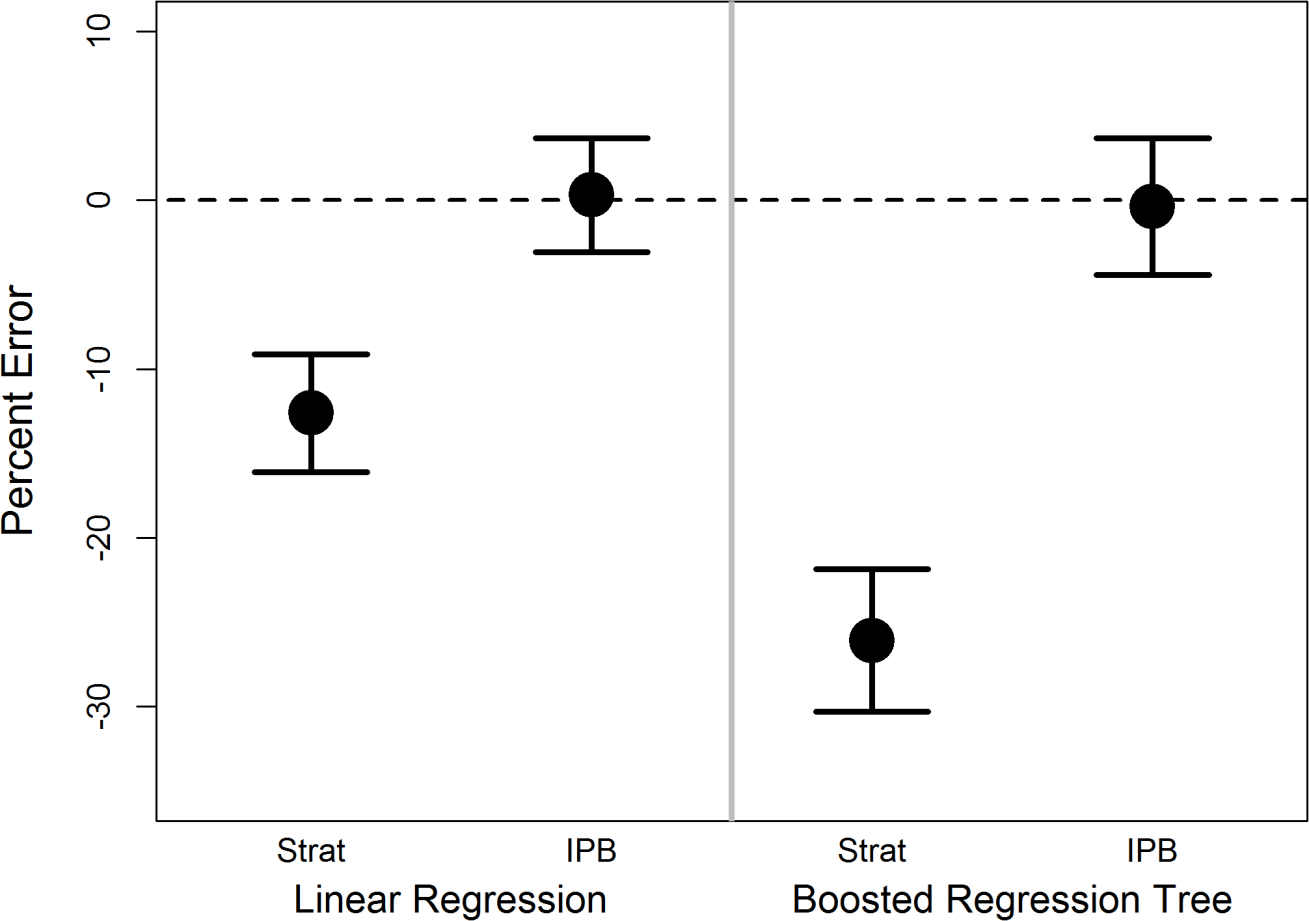
Mean and 95% confidence intervals for cross validation prediction error for regression of steelhead density on independent variables, and boosted regression tree analysis of steelhead density, as a percentage of the mean observed steelhead density at all sites. Models are built on data from stratified sample ignoring sample inclusion probabilities (Srat), and Inverse Probability Bootstrap samples (IPB)

Cross validation results from linear regression and boosted regression tree analysis showed biased predictions, for both models, when sample inclusion probabilities were ignored, while IPB sampling resulted in unbiased estimates (Fig 6 and Table 8). Biases after IPB sampling were consistently near zero. Precision of model predictions, estimated as the standard deviation of the cross validation errors, did not differ greatly between the two methods.

**Table 8:** Cross validation results for example 3: bias and standard deviation of predicted-measured ln(Steelhead per m^2^).

	Linear Regression on Selected CHaMP Metrics		Quantile Regression on Selected CHaMP Metrics		Boosted Regression Tree	
	Bias	Std. Dev.	Bias	Std. Dev.	Bias	Std. Dev.
**Stratified Sample, Ignoring Inclusion Probability**	-0.23	0.61	0.05	0.56	0.42	0.70
**Inverse Probability Bootstrap Sampling**	-0.01	0.61	0.00	0.60	-0.01	0.71

When comparing coefficients for linear and quantile regression models, we assumed that IPB sampling provided approximately unbiased estimates and that the differences between non-IPB sampling and IPB sampling therefore approximated biases resulting from ignoring sample inclusion probabilities. For linear regression, percent errors in the nine coefficient estimates when sample inclusion probabilities were ignored ranged from 14% to 89% (Table 9). For quantile regression, percent errors in coefficient estimates ranged from 12% to over 500%, with 3 of 8 parameter estimates having errors or more than 100% (Table 10).

**Table 9:** Parameter estimates for example 3, regression of ln(steelhead density, fish/m^2^) on selected habitat parameters, for models that: ignore sample inclusion probabilities, and utilize IPB sampling to account for sample inclusion probabilities.

	Stratified Sample: Inclusion Probabilities Ignored in Model Fitting Process		Inverse Probability Bootstrap		% Error Due to Ignoring Weights
Parameter	Est. Slope	Std. Error	Est. Slope	Std. Error	
**Intercept**	-1.60	0.027	-1.49	0.031	-7%
**Conductivity**	0.13	0.030	0.23	0.023	46%
**Site Bankfull Area**	-0.35	0.111	-0.68	0.137	49%
**Wetted Large Wood Volume By Site**	-0.01	0.034	-0.13	0.038	89%
**Fast Non-Turbulent Area**	-0.05	0.029	-0.09	0.038	39%
**Mean Bankfull Width Mean**	0.19	0.106	0.52	0.138	64%
**Boulders**	0.09	0.027	0.13	0.028	36%
**Fish Cover Composition LWD**	-0.04	0.036	-0.08	0.028	44%
**Site Discharge**	-0.06	0.031	-0.07	0.043	15%
**Fines <2mm**	0.06	0.036	0.07	0.036	14%

Standard errors refer to the parameter estimates.

**Table 10:** Parameter estimates for example 3, comparison for 95^th^ percentile quantile regression of ln(steelhead density, fish/m^2^) on selected habitat parameters, for models that: ignore sample inclusion probabilities, and utilize IPB sampling to account for sample inclusion probabilities.

	Weights Ignored in Model Fitting Process		Inverse Probability Bootstrap		% Error Due to Ignoring Weights
Parameter	Est. Slope	Std. Error	Est. Slope	Std. Error	
**Intercept**	-0.66	0.08	-0.61	0.08	-9%
**Conductivity**	0.25	0.07	0.31	0.07	19%
**Site Bankfull Area**	-0.68	0.35	-1.25	0.38	45%
**Wetted Large Wood Volume By Site**	0.06	0.09	0.07	0.12	15%
**Fast Non-Turbulent Area**	-0.07	0.10	0.02	0.08	538%
**Mean Bankfull Width**	0.39	0.32	0.96	0.36	59%
**Boulders**	0.04	0.08	-0.03	0.11	220%
**Fish Cover Composition LWD**	-0.07	0.11	-0.21	0.06	64%
**Site Discharge**	-0.10	0.08	-0.12	0.12	12%
**Fines <2mm**	0.03	0.08	-0.13	0.07	124%

Standard errors refer to the parameter estimates.

Interestingly, estimates of bias induced by ignoring sample inclusion probabilities in quantile regression were, on a percentage basis, higher than those obtained from linear modeling, suggesting that quantile regression of fish abundance for CHaMP/ISEMP data may be more sensitive to bias induced by ignoring sample probabilities than is ordinary regression. Similarly, bias observed in boosted regression tree analysis was also greater than bias induced by linear regression, when sampling design was ignored in the analysis.

## Discussion

Like conventional bootstrapping, inverse probability bootstrapping is a technique that can broadly be applied and, like conventional bootstrapping, is easily accessible to ecological researchers. It can be incorporated into model based analyses of data from complex, unequal probability samples common in ecological research.

Our results confirmed that estimates from model based analyses of data obtained from unequal probability samples are susceptible to sample selection bias if sample inclusion probabilities are ignored. Because stratified sampling and other forms of unequal probability sampling are useful and common in the collection of ecological data, and because ecological datasets may have complicated variance-covariance structures with known and unknown correlations among variables, analyses of ecological datasets are particularly susceptible to such bias. Sample selection bias, unlike standard error, does not tend toward zero as sample size increases [10], nor is bias reported as an output of statistical tools. It is incumbent on the researcher to recognize potential bias, and avoid misconstruing equality of reported standard errors and evidence of equally valid analyses.

By re-sampling in a manner that creates an equal probability sample from the original sample rather than relying on modification or augmentation of model based analysis tools, we greatly expand the number of model based tools available for the analysis of complex ecological survey designs. Sufficient iteration of the re-sampling process ensures that essentially all information content in the original probability sample is retained. Thus there is no appreciable loss in precision of estimates, but rather only an elimination of sample induced bias. While the intended analysis should, whenever possible, be considered at the point of sampling design, it is hoped that in pursuit of evolving objectives, or in the interest of taking full advantage of existing datasets, study design need not limit the choice of statistical modeling tools considered.

We expected standard error estimates based on IPB sampling to be reasonably accurate, and observed that the standard error estimates in analysis of IPB samples were similar to the standard errors estimated using unequal probability samples while ignoring sample inclusion probabilities. Further study is needed to assess the accuracy and statistical consistency of standard error estimates based on IPB sampling, either theoretically or through simulation. Currently, we recommend reliance on cross validation techniques to assess model precision. While uncertainty in standard error estimates is a potential limitation of IPB bootstrapping, this limitation is tempered significantly if one accepts that it is also advisable to use cross validation whenever possible, regardless of whether one is utilizing IPB or not, in the assessment of statistical models in ecology. There are numerous cross validation approaches are available, even for datasets with small sample sizes [33, 34].

While stratified samples and other forms of unequal probability samples are common in ecological research, it is also becoming more common to perform analyses on datasets built from aggregating two or more samples. In any aggregate dataset, even when simple random sampling is used for each original dataset, aggregate datasets are likely to have unequal sample inclusion probabilities due to differences in sampling frames and sampling densities in the original samples. *Earth Cube*, for example, is a National Science Foundation sponsored program aimed at providing “unprecedented data across the geosciences” (earthcube.org). Providing aggregate datasets obtained from multiple sources is an obvious objective of such programs. CHaMP/ISEMP data are also publically available, and researchers may wish to merge CHaMP/ISEMP data with other sources of data to form aggregate datasets. While caution should be used in aggregation of data from multiple sources, the generation of unequal sample inclusion probabilities need not limit the statistical tools or defining statistical scope of inference. Sample inclusion probabilities for aggregate datasets can be calculated, provided that the sample inclusion probabilities of the original datasets are known. Gathering adequate metadata to enable estimation of sample inclusion probabilities is an obvious challenge in the use of aggregate datasets, but in instances where this is available and complex model based tools are required, IPB is a powerful tool enabling the appropriate incorporation of sample design probabilities to overcome challenges in unequal probability samples.

In some instances, datasets may be available to researchers, but metadata required to estimate sample inclusion probabilities may not be available. Given the susceptibility to bias when sample inclusion probabilities are not accounted for in an analysis, caution and transparency should be exercised. In some cases, methods such as raking [35] or post stratification [22] have been used in order to estimate sample inclusion probabilities from the data itself [36]. Such estimated sample inclusion probabilities can also be used, with caution, with IPB sampling or other statistical tools that account for sample inclusion probabilities.

## References

[1] Ioannidis JPA (2005). Why most published research findings are false. PLoS Med 2(8): e124 10.1371/journal.pmed.0020124 16060722 PMC1182327

[2] Thomas SL, Heck RH, 2001 Analysis of Large-Scale Secondary Data in Higher Education Research: Potential Perils Associated with Complex Sampling Designs, Research in Higher Education, Vol 42, Issue 5, p 517–540.

[3] Stevens DL Jr, and Olsen AR (2004). Spatially balanced sampling of natural resources. J Am Stat Assoc, 99:465, 262–278, DOI: 0.1198/016214504000000250.

[4] George BJ, Sobus JR, Phelphs LP, Rashleigh B, Simmons JE, Hines RN, et al. (2015). Raising the Bar for Reproducible Science at the U.S. Environmental Protection Agency Office of Research and Development. Toxicological Sciences, March2015, 1–7 10.1093/toxsci/kfv020 PMC4408961 25795653

[5] Howes S, and Lanjouw JO (2005). Does Sample Design Matter for Poverty Rate Comparisons? The Review of Income and Wealth, Vol 44 issue 1, p 99–109.

[6] Goodman KJ, Parker SM, Edmonds JW, Zeglin LH (2015). Expanding the Scale of Aquatic Sciences: the Role of the National Ecological Observatory Network (NEON). Freshwater Sciences, Vol. 34, No. 1, pp377–385.

[7] Kozak M, Zielinski A (2007). Comparison of efficiency of stratified and unequal probability sampling. Commun Stat Simul Comput. pp.807-816, 10.1080/03610910701419695

[8] Valliant R, Dorfman AH, Royall RM (2000). Finite population sampling and inference: a prediction approach. Wiley, New York, NY, USA.

[9] Jongman RHG, Bunce RHG, Metzger MJ, Mücher CA, Howard DC, et al (2006). Objectives and applications of a statistical environmental stratification of Europe. Landsc Ecol 21:409–419.

[10] Lohr S (1999). Design and analysis, Duxbury Press, ISBN 0-534-35361-4.

[11] Phillips SJ, Dudík M, Elith J, Graham CH, Lehmann A, Leathwick J, et al (2009). Sample selection bias and presence-only distribution models: implications for background and pseudo-absence data. Ecol Appl: 19: 181–197. 19323182 10.1890/07-2153.1

[12] Fancy SG, Gross JE, Carter SL (2009). Monitoring the condition of natural resources in US national parks. Environ Monit Assess 151,161–174. 10.1007/s10661-008-0257-y 18509737

[13] Ward MB, Nelle P, Walker SM. (editors) (2011). CHaMP: 2011 pilot year lessons learned project synthesis report. Prepared for the Bonneville Power Administration by CHaMP Published by Bonneville Power Administration, Portland, OR. 95 pages.

[14] Reynolds JH (2012). An overview of statistical considerations in long-term monitoring. Design and analysis of long-term ecological monitoring studies, Cambridge University Press ISBN 978-0-521-13929-8.

[15] R Core Team (2014). R: A language and environment for statistical computing. R Foundation for Statistical Computing, Vienna, Austria URL http://www.R-project.org/.

[16] Kincaid TM, Olsen AR (2013). Spsurvey: spatial survey design and analysis. R package version 2.6. URL http://www.epa.gov/nheerl/arm/.

[17] Gregoire TG (1998). Design-based and model-based inference in survey sampling: appreciating the difference. Can J For Res. 10.1139/x98-166

[18] De Gruijter JJ, Brus DJ, Bierkens MFP, Knotters M (2006). Sampling for natural resource monitoring. Springer Publishing, New York, NY, USA.

[19] Strand EK, Bunting SC, Nahorniak, MT, Starcevich LAH (2013). Upper Columbia basin network aspen monitoring report 2008–2012: City of Rocks National Reserve (CIRO). Natural Resource Technical Report NPS/UCBN/NRTR—2013/795. National Park Service, Fort Collins, Colorado.

[20] Chamber R.L., Clark R.G, 2012 An introduction to Model based survey design with applications New York: Oxford University Press.

[21] Lumley T (2004). Analysis of complex survey samples. J Stat Softw 9(1): 1–19.

[22] Lumley T (2012). Survey: analysis of complex survey samples. R package version 3 28–2.

[23] Skare Ø, Bølviken E, Holden L (2003). Improved Sampling-Importance Resampling and Reduced Bias Importance Sampling. Scandinavian Journal of Statistics, 30: 719–737. 10.1111/1467-9469.00360

[24] Efron B (1979). Bootstrap methods: another look at the jackknife. Ann Stat 7 no. 1, 1–26. 10.1214/aos/1176344552 URL http://projecteuclid.org/euclid.aos/1176344552.

[25] Rao JNK, Wu CF J (1988). Resampling inference with complex survey data. J Am Stat Assoc 83231–241.

[26] Sitter RR (1992). A resampling procedure for complex survey data. J Am Stat Assoc 87(419).

[27] Walesiak M (2014). ClusterSim: searching for optimal clustering procedure for a data set. R package version 0.43–4.

[28] Girvetz EH (2007). Multi-scale habitat patch modeling: integrating landscape pattern, habitat suitability and population dynamics with implications for ecology and conservation, Ph.D. Thesis. University of California, Davis.

[29] Cade BS, Terrell JW, Schroeder R (1999). Estimating effects of limiting factors with regression quantiles. Ecology, 80(1):311–323

[30] Haire S, Bock C, Cade B, Bennett B (2000). The role of landscape and habitat characteristics in limiting abundance of grassland nesting songbirds in an urban open space. Landscape and Urban Planning, 48(1):65–82

[31] Elith, J, Leathwick, JR, and Hastie T (2008). Boosted regression trees–a new technique for modelling ecological data. Journal of Animal Ecology. 10.1111/j.1365-2656.2008.01390.x 18397250

[32] Elith J, Leathwick JR, Hastie T (2008). A working guide to boosted regression trees, Journal of Animal Ecology 77, 802–813. 10.1111/j.1365-2656.2008.01390.x 18397250

[33] Mayer DG, Butler DG (1993). Statistical validation. Ecological Modeling, 68:21–32.

[34] Sylvain A, Alain C (2010). A survey of cross-validation procedures for model selection. Statistics Surveys, 4, (2010), 40–79 (electronic). 10.1214/09-SS054

[35] Deville JC, Särndal CE and Sautory O (1992). Generalized raking procedures in survey sampling. Journal of the American Statistical Association, Vol. 88, Issue 483.

[36] Lennet-Cody CE, Buckland ST, and Marques FFC (2001). Trends in dolphin abundance estimated from fisheries data: a cautionary note. Journal of Cetacean Research and Management 3:305–319

